# Supplementary material for: Capacitive tendency concept alongside supervised machine-learning toward classifying electrochemical behavior of battery and pseudocapacitor materials
Source: Nat Commun. 2024 Feb 7;15:1133. doi: 10.1038/s41467-024-45394-w (PMC10850137; doi:10.1038/s41467-024-45394-w)
Supplement: Supplementary file 1 — Supplementary Information [file 41467_2024_45394_MOESM1_ESM.pdf]

Supplementary Information

Capacitive tendency concept alongside supervised machine-learning toward classifying electrochemical behavior of battery and pseudocapacitor materials

Sirapprapha Deebansok,[1] Jie Deng,[2] Etienne Le Calvez,[3,4] Yachao Zhu,[5] Olivier Crosnier,[3,4] Thierry Brousse,[3,4] Olivier Fontaine[1,6✉]

[1] Molecular Electrochemistry for Energy laboratory, VISTEC, Institute of Science and Technology, Rayong, 21210, Thailand.

[2] Institute for Advanced Study & College of Food and Biological Engineering, Chengdu University, Chengdu 610106, China.

[3] Nantes Université, CNRS, Institut des Matériaux de Nantes Jean Rouxel, IMN, 44000 Nantes, France.

[4] Réseau sur le Stockage Électrochimique de l'Énergie (RS2E), CNRS FR 3459, 33 rue Saint Leu, 80039 Amiens, France.

[5] ICGM, Université de Montpellier, CNRS, 34293 Montpellier, France.

[6] Institut Universitaire de France, 75005 Paris, France.

✉ Corresponding author. Email: Olivier Fontaine: olivier.fontaine@vistec.ac.th

Table of Contents

1. The architectures for classification and image recognition ... 3

1.1 ResNet50 ... 3
1.2 Xception ... 3
1.3 VGG-16 ... 4
1.4 MobileNetV2 ... 4
1.5 8-layer CNN ... 4

2. Evaluations ... 6

3. Data collection, model training, and classification ... 8

4. Training dataset ... 10

5. Loss curves ... 10

6. Machine-learning for *CV*/*GCD* classification ... 16

7. Code and dataset accessibility on Github repository ... 18

8. The website description ... 21

8.1 Home page ... 21
8.2 T.B Robot page ... 24
8.2.1 *GCD* classification page ... 26
8.2.2 *CV* classification page ... 27
8.3 Special issues ... 28
8.3.1 *GCD* classification ... 28
8.3.2 *CV* classification ... 31

9. The comparison of literatures of the capacitive prediction ... 34

Supplementary References ... 36

1. The architectures for classification and image recognition

1.1 ResNet50

Residual Network (ResNet) was built by Kaiming He et al. for the competition in the ILSVRC (ImageNet Large Scale Visual Recognition Challenge) 2015 challenge, in 2015. ResNet has many variants that run on the same concept but have different numbers of layers. Resnet50 is denoted as ResNet architecture with 50 neural network layers. The advantage of this architecture is the ability to train a deep network by skip connections (also called shortcut connections) when the output of the previous layer is injected into the deeper layer as illustrated in **Supplementary Figure 1.**[1]

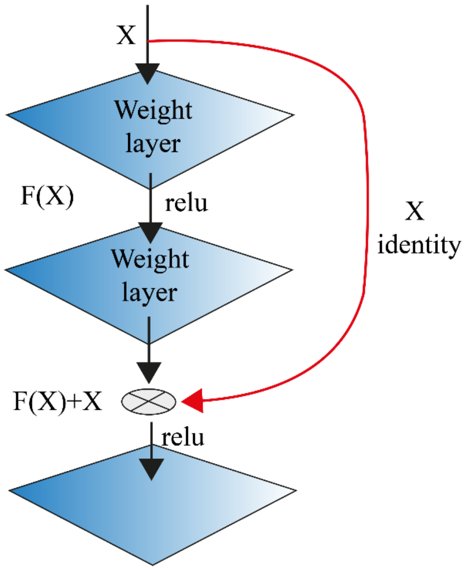

Supplementary Figure 1 | Residual learning; a building block.[1]

1.2 Xception

Xception or Extreme version of Inception is a convolutional neural network that contains 71 layers deep developed by Google. With a modified depthwise separable convolution, it is more efficient

than Inception-v3 (also by Google, 1st Runner Up in ILSVRC 2015) for both ImageNet ILSVRC and JFT datasets.[2]

1.3 VGG-16

VGG-16 is a convolutional neural network containing 16 layers deep that won the ILSVR(Imagenet) competition in 2014. This model can classify images as 1000 different types even with the pretrained network. VGG-16 architecture is composed of 16 convolution layers and 3 fully connected layers.[3]

1.4 MobileNetV2

MobileNetV2 is based on an inverted residual structure where the thin bottleneck structure in the model. This thin bottleneck is the input and output of the residual blocks compared to the conventional in which the expanded input is used. The model uses 3×3 depthwise separable convolutions. Consequently, the smaller computational cost than that of standard convolutions.[4]

1.5 8-layer CNN

The component of a CNN contains three main types of layers including pooling, convolution, and the fully connected layers. The features can be extracted by pooling, convolution, while the fully connected block provide the output when the extracted features is mapped on the output as shown in **Supplementary Figure 2**.[5]

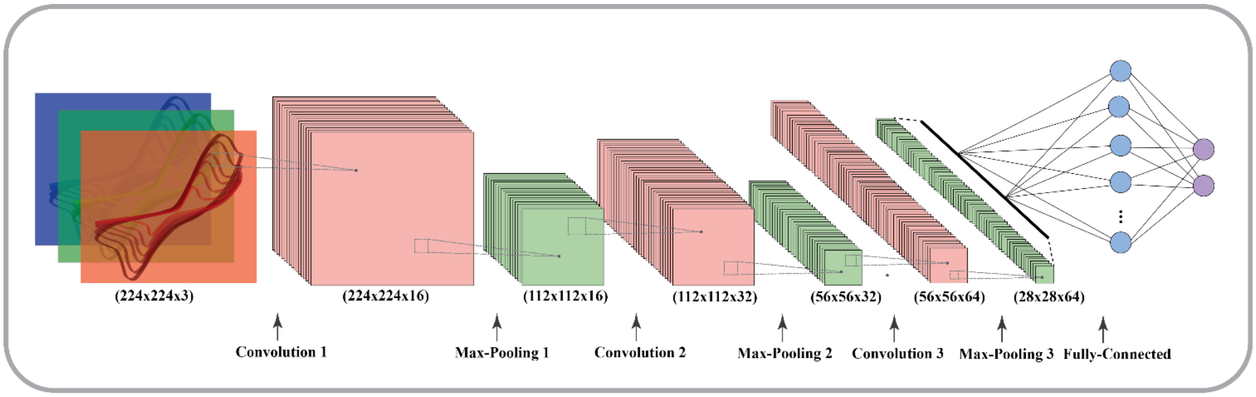

Supplementary Figure 2 | The convolutional layers of 8-layer CNN architecture.[5]

1.5.1. Convolutional layer

This layer is where the feature extraction takes place by the linear or non-linear operation. Here, kernel or filter that act as the operator applies to the input. The type of input is the image which will be transformed into the arrays of number called tensor.[5]

1.5.2. Pooling layer

Pooling layer is the layer in CNN where reduced a matrix (or matrices) is created to reduce the dimensionality of the feature matrix. Pooling normally composed of filter size, stride, and padding operator. It also includes taking either the maximum or average value across the pooled area. This layer subsequently deceases the learnable parameter.

1.5.3. Fully connected layers

A fully connected layer is also known as a dense layer. It is a hidden layer in which each node of the input is connected to every node of the output in the subsequent hidden layer and followed by the non-linear function, for example, ReLU (Rectified linear unit).[5]

2. Evaluations

The evaluation values were calculated as Accuracy, Sensitivity, Specificity, Precision, and F-Score, which describes in the following part based on the confusion matrix as shown **in Supplementary Figure 3**.

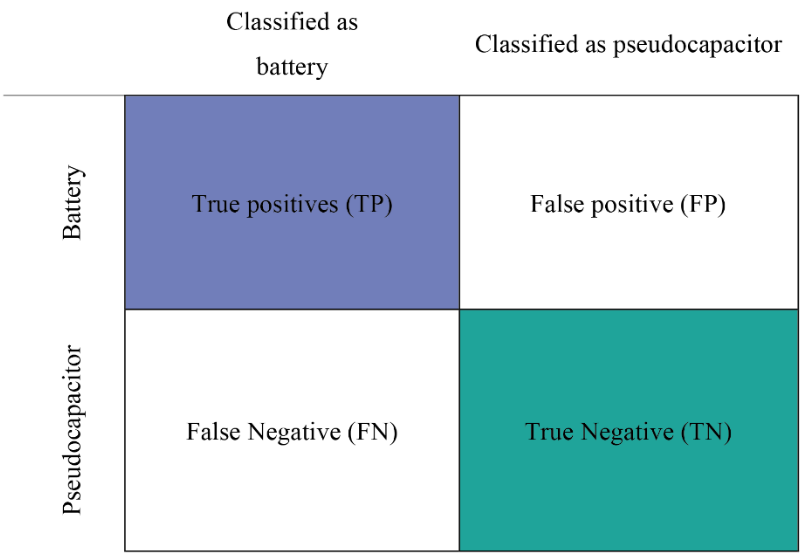

Supplementary Figure 3 | Confusion matrix. True Positive, False Positive, False Negative, and True Negatives are defined based on the classification results (classified battery and classified pseudocapacitor) and the set of references (battery and pseudocapacitor)

$$\text{Accuracy} = \frac{\text{TP+TN}}{\text{TP+TN+FP+FN}} \qquad \textbf{(S1)},$$

where accuracy is the ratio of correctly predicted observation (TP+TN) compared to the total observations (TP+TN+FP+FN),

$$\text{Precision} = \frac{\text{TP}}{\text{TP+FP}} \qquad \textbf{(S2)},$$

where precision is the fraction of relevant instances (TP) among the retrieved instances (TP+FP),

$$\text{Sensitivity} = \frac{\text{TP}}{\text{TP+FN}} \qquad \textbf{(S3)},$$

where recall/sensitivity (SE) is the fraction of relevant instances on battery that have been retrieved over total relevant instances in the image. It is based on an understanding and measuring of relevance,

$$\text{Specificity} = \frac{\text{TN}}{\text{TN+FP}} \qquad \textbf{(S4)},$$

where specificity (SP) is the fraction of relevant instances on pseudocapacitor that have been retrieved over total relevant instances in the image. It is based on an understanding and measuring of relevance,

$$\text{F1-Score} = \frac{\text{2譚P}}{\text{2譚P+FP+FN}} \qquad \textbf{(S5)},$$

where F1-score is harmonic mean of precision and recall. As a result, a high F1-score can only be obtained with both high recall and high precision are high.[6]

3. Data collection, model training, and classification

The first step was to extract the figures from the scientific paper using the PyMuPDF library in Python. Each figure could contain multipleCVs or GCDs. The OpenCV library was then used to separate each CV or GCD image. The resulting dataset contained CVs, GCDs, and other images, such as the author's image, the journal's logo, and illustrations. This is illustrated in **Supplementary Figure 4**.

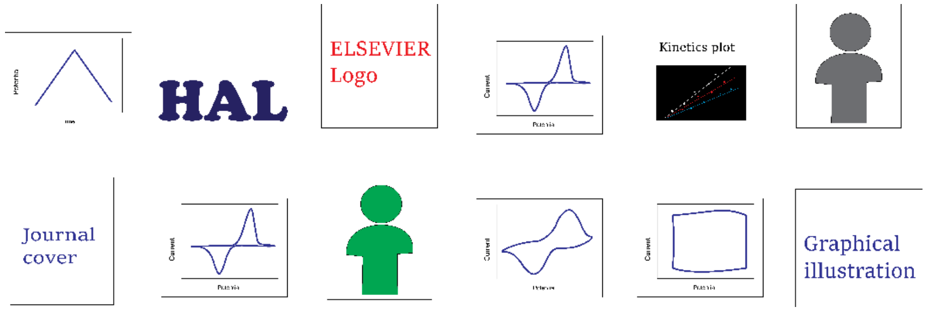

Supplementary Figure 4 | The representative of images extracted from the article.

Since the number of images extracted from the articles was large, we needed to screen and collect only the CVs and GCDs from the entire image dataset. To do this, we quickly classified the extracted images using the ResNet50 model, which only collects CV and GCD images (this step is called Process 1). This is illustrated in Supplementary Figure 5. Firstly, the images of CV and GCD were manually labelled by human to be used as the training dataset for Process 1 (distinguishing CV/GCD from the other images). Process1 was then performed (using ResNet50 architecture) to classify and collect only CV and GCD images from all unclassified images. The prediction (Output1) will finally compose of 3 categories:

1. CV
2. GCD

3. Other images.

Here, only the CV and GCD were then used for the classification in the next step (Process2, 3, 4, and 5) later.

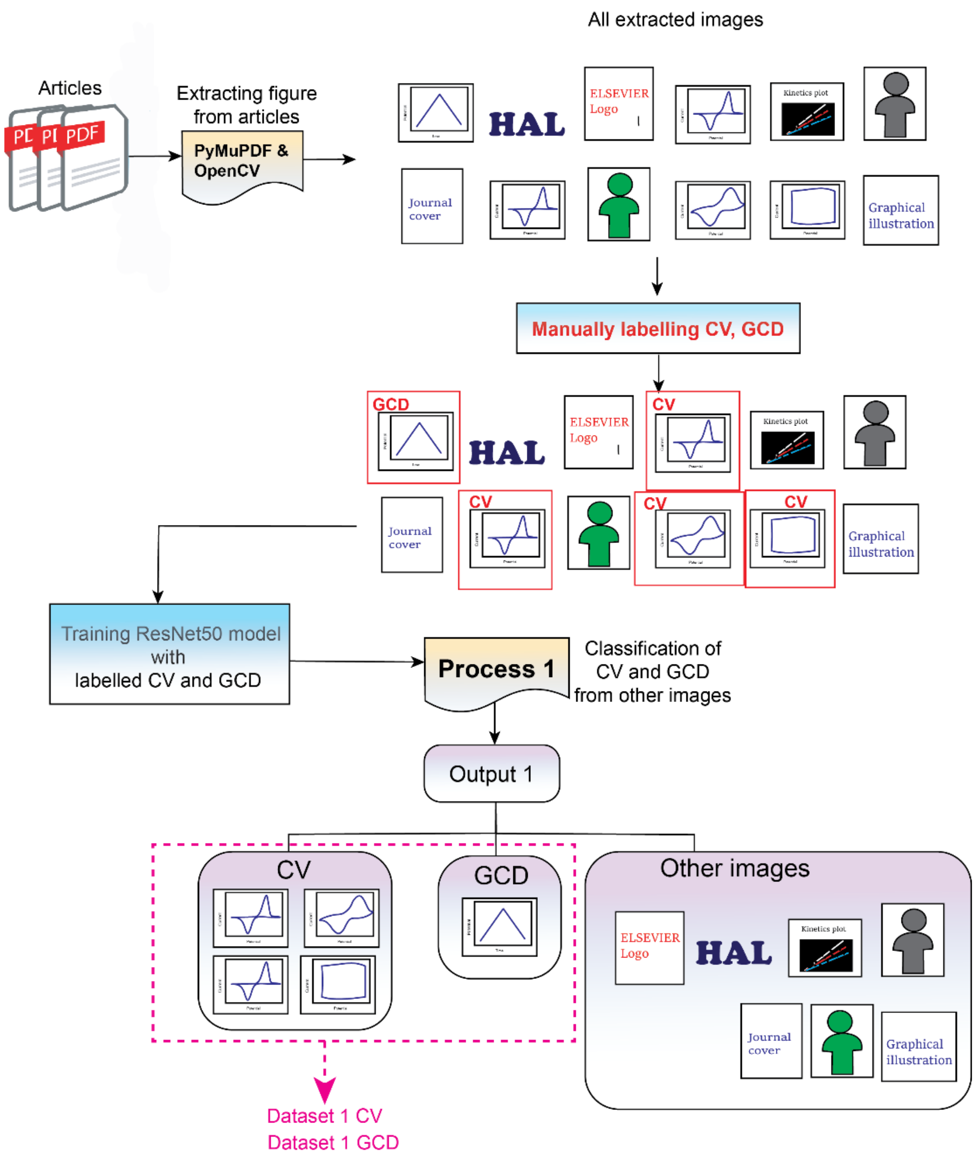

Supplementary Figure 5 | Process 1: Extraction and collection of CV and GCD images from the articles.

4. Training dataset

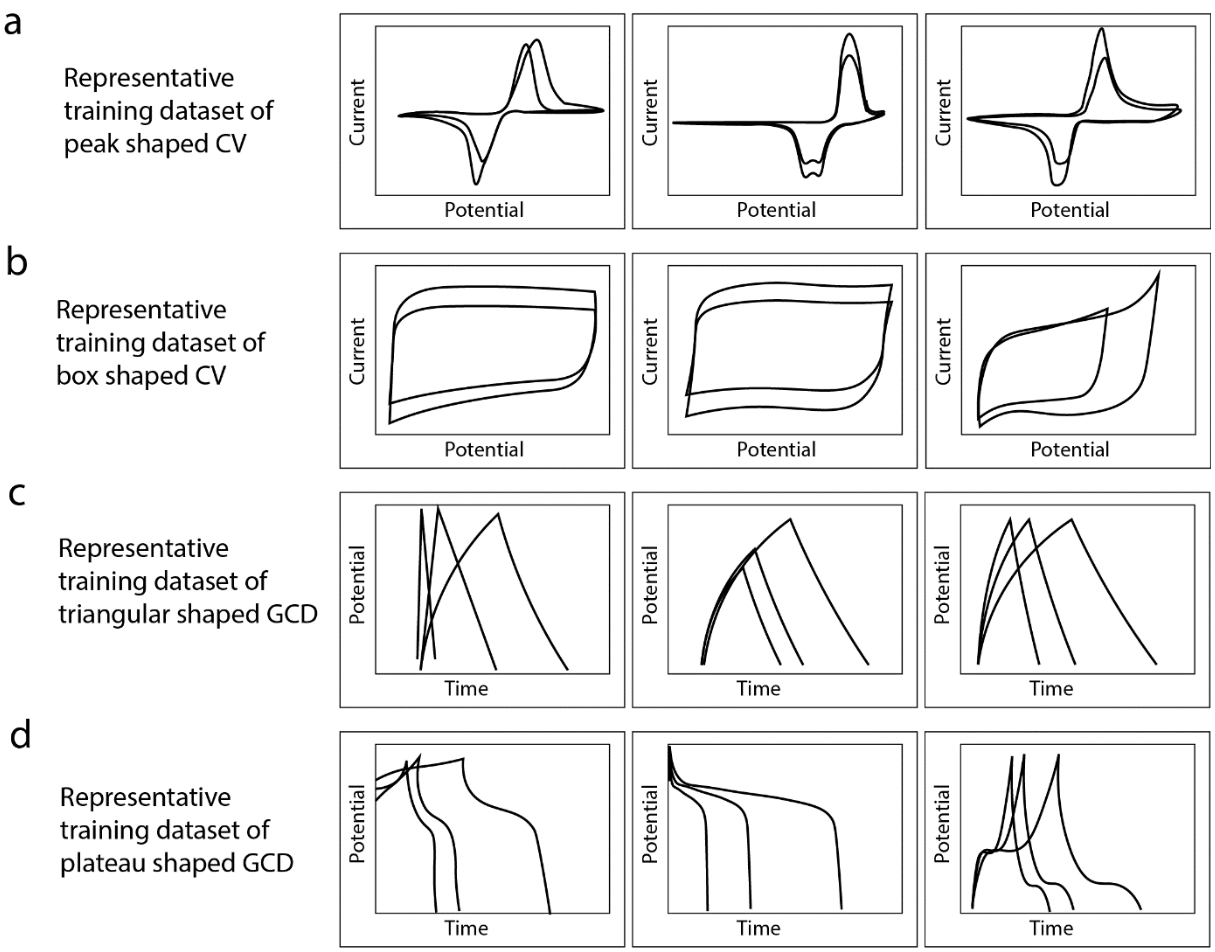

Supplementary Figure 6 | The representative of training datasets of CV (a) box, (b) peak characteristic, and GCD (c) triangular, and (d) plateau characteristic.

5. Loss curves

Training loss and validation loss were plotted in order to determine the number of loop that optimized the prediction with highest accuracy and lowest Loss Value.[7] In this work, the loss

curves (**Supplementary Figure 7-11**) were used to validate the best architecture to be used for the classification with our datasets.

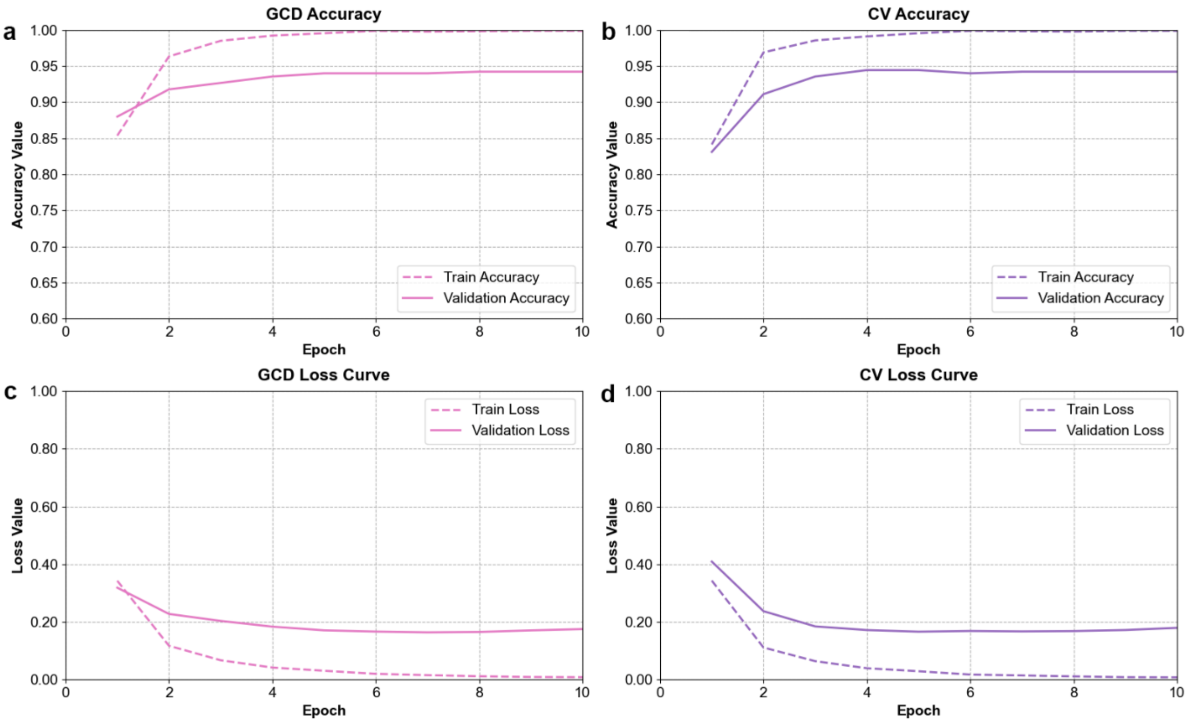

Supplementary Figure 7 | Loss curves of the training and validation loss for *GCD* and CV classification by using model ResNet50.

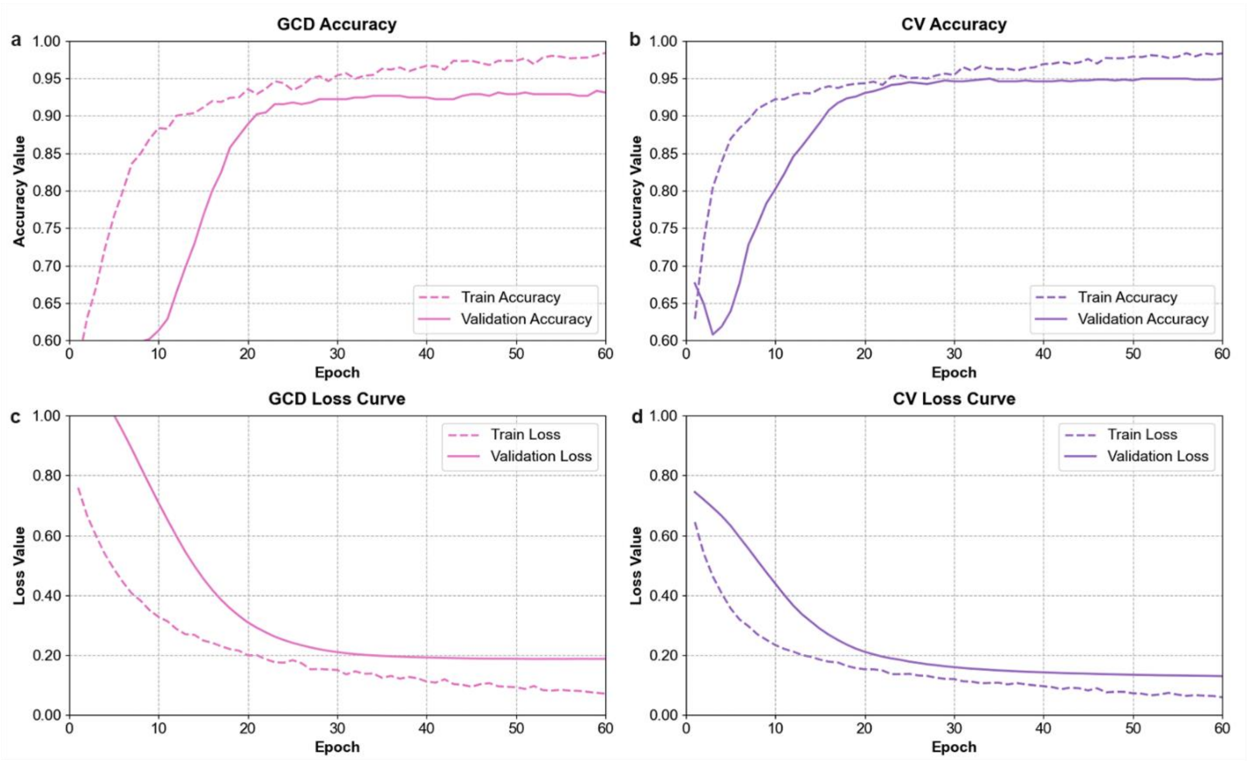

Supplementary Figure 8 | Loss curves of the training and validation loss for *GCD* and CV classification by using MobileNetV2 architecture.

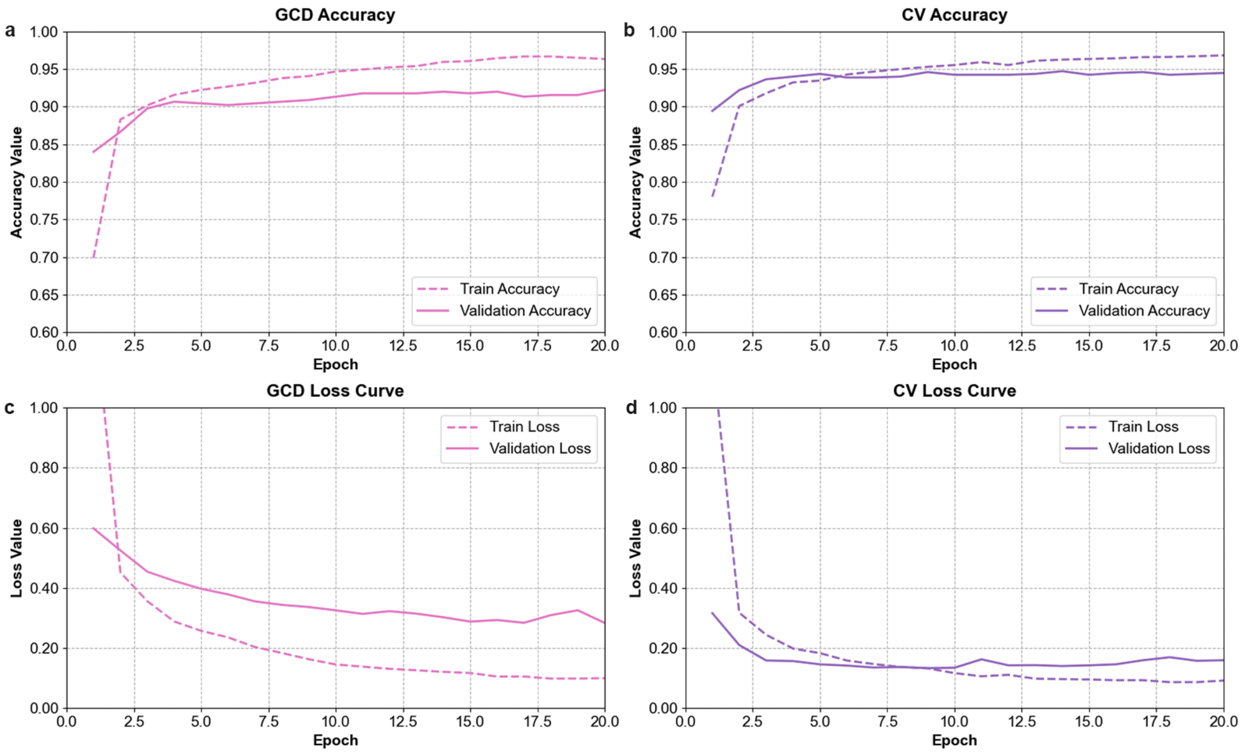

Supplementary Figure 9 | Loss curves of the training and validation loss for *GCD* and CV classification by using VGG16 architecture.

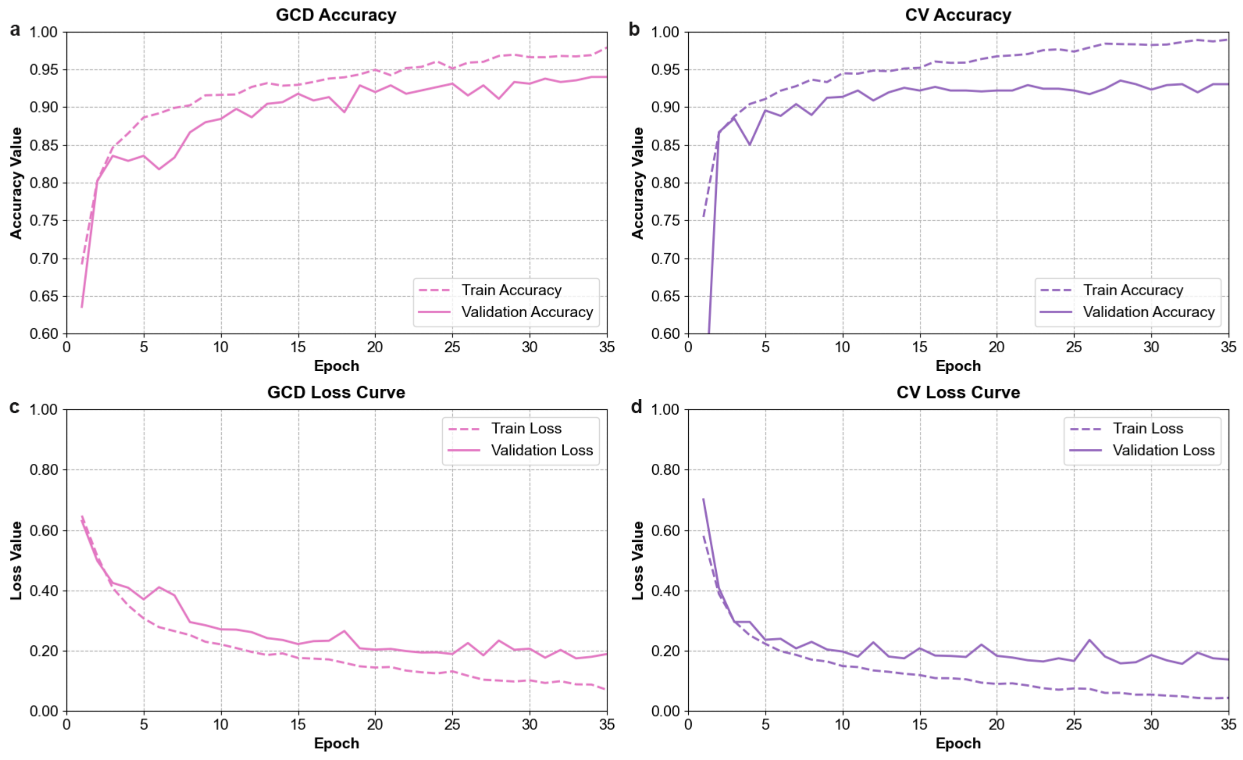

Supplementary Figure 10 | Loss curves of the training and validation loss for *GCD* and CV classification by using Xception architecture.

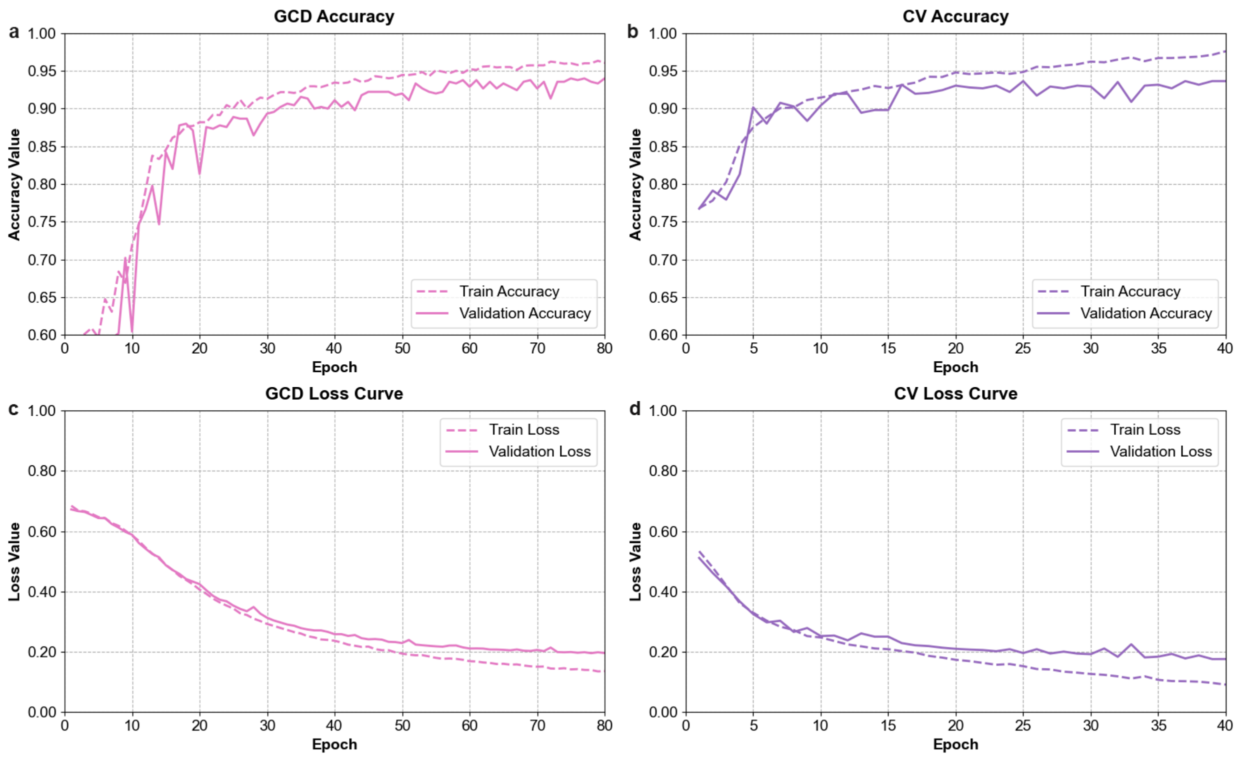

Supplementary Figure 11 | Loss curves of the training and validation loss for *GCD* and CV classification by using 8-Layer CNN architecture.

6. Machine-learning for *CV*/*GCD* classification

Supplementary Table 1 | The models used with various sets of inputs and outputs based on ResNet50 architecture.

Process	Input	Output	Output data
**1**	Image	1	Other image, *CV*, *GCD* (%confidence)
**2**	*GCD*	2	Battery, Pseudocapacitor (%confidence)
**3**	*CV*	3	Battery, Pseudocapacitor (%confidence)
**4**	*CV* of Battery	4	Capacitive tendency (0-50 %)
**5**	*CV* of Pseudocapacitor	5	Capacitive tendency (50-100 %)

Supplementary Table 2 | *GCD* and *CV* classification comparison based on evaluation values obtained from five different architectures; *ResNet50*, *MobileNetV2*, *VGG16*, *Xception*, and *8-Layer CNN*.

CNN-Model	Accuracy (%)	Sensitivity (%)	Specificity (%)	Precision (%)	F1 -Score
GCD					
ResNet50	94.22	93.84	94.45	94.16	93.99
MobileNetV2	93.11	92.56	93.07	93.12	92.82
VGG16	92.22	92.24	94.61	91.78	91.99
Xception	93.77	93.98	96.49	93.33	93.60
8-Layer CNN	94.00	93.73	94.77	93.82	93.78
CV					
ResNet50	95.80	93.52	96.74	94.65	94.07
MobileNetV2	94.64	92.62	96.42	93.24	92.92
VGG16	94.36	93.12	97.13	91.53	92.28
Xception	93.04	88.87	94.35	91.31	90.00
8-Layer CNN	93.65	89.08	94.26	92.77	90.74

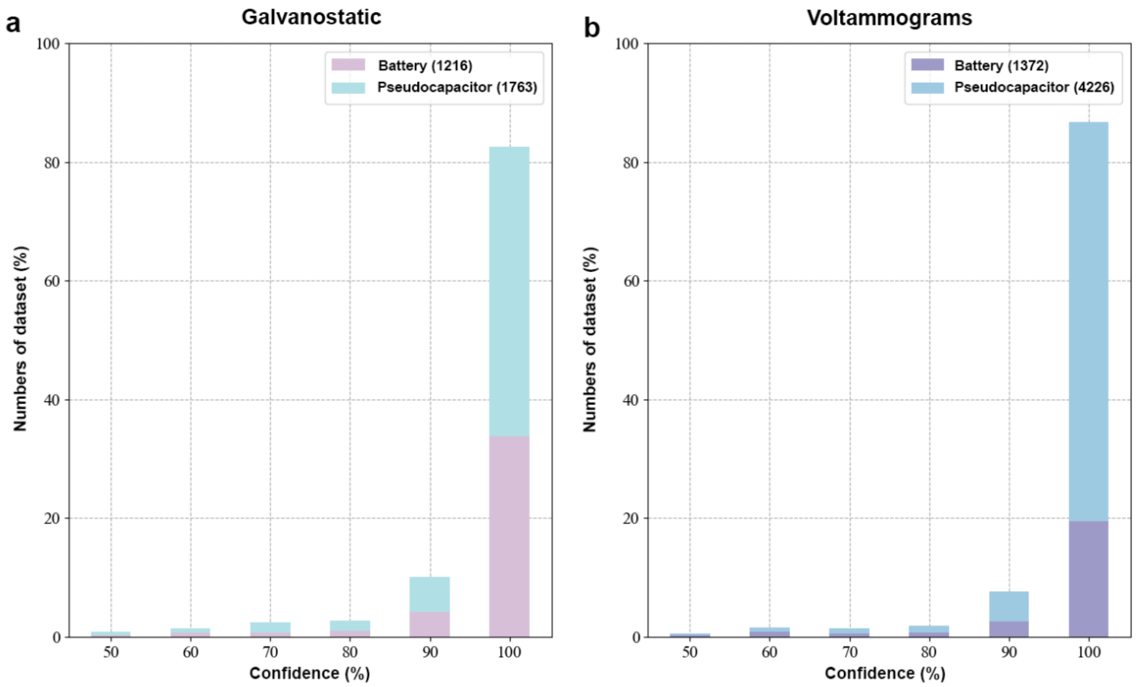

Supplementary Figure 12 | The distribution of classification efficiency on a) *GCD*, and b) *CV* classification using Process 2, and 3, respectively.

Supplementary Figure 9 shows the results of classification using ResNet50 architecture with two type of input data including GCDs (Supplementary Figure 9a) and CVs (Supplementary Figure 9b). It shows percentage of input CV/GCD images (y-axis) were classified as battery or pseudocapacitor with respect to the percentage of confidence (x-axis). The result shows that more than 80% of testing input was classified with 100% confidence.

7. Code and dataset accessibility on Github repository

To access and run scripts with our codes and datasets, user can clone out repository from Github following this instruction:

7.1 Clone the repository

By going to the website (https://github.com/ice555mee/TB-robot_code-data) or using this command

```
git clone https://github.com/ice555mee/TB-robot_code-data.git
```

7.2 Repository branches

This project has two branches: **main, master**, which can be explained here

- **Main**: contains aggregate codes and datasets
- **Master**: additionally contains codes that user can use for test running. All models (including file.py, file.h5, file.json) are located in master branch of this repository since the large files are only store in master branch.

7.3 Installation/running

You may need to install the libraries/packages according to Python requirments.txt file.

- Code location

Codes for prediction test are located in folder named '**Test_prediction**' in main branch, where all model files are located in master branch following to the structure below:

```
Main branch
└── Test_prediction
    ├── CV_classification.py
    ├── GCD_classification.py
    └── requirements.txt

Master branch
└──Test_prediction
```

- ○ File named ‘**CV_classification.py**’ or ‘**GCD_classification.py**’ are for the classification test for CV and GCD, respectively.
- ○ File named ‘**requirements.txt**’ contains all requirements of packages and versions to be installed.

- Install the requirements by this following command

```
pip install -r requirements.txt
```

- Run prediction
  - ○ Run files named ‘**CV_classification.py**’ or ‘**GCD_classification.py**’
  - ○ Before running, edit file paths for the testing images (line 49) and the directory (line 51), where the user wants to put the results:

```
48
49  image_path = r'C:\Users\ice_d\Desktop\Python\Test_prediction\unknownCV\unknown1.jpeg'
50  # new Image directory
51  newdirec= r'C:\Users\ice_d\Desktop\Python\Test_prediction\resultCV'
52  newloca=os.chdir(newdirec)
53  # New filename
54  newfilename = 'predicted'
55
```

- Results

The result will show in the terminal with percentage confident of {type of material as Battery or pseudocapacitor}. For example,

```
1/1 [==============================] - 0s 106ms/step
confident 0.9916572570800781 of  {'Pseudocapacitor'}
finished prediction
```

, while the predicted CV or GCD images will be saved in the location as programed as shown below.

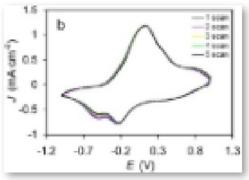

predicted_Pseudocapacitor_confident 0.9916572570...

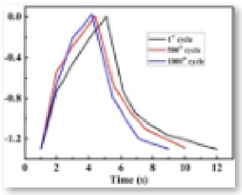

predicted_1_Pseudocapacitor_0_0.9561004638671875

8. The website description

In this work, the website (http://supercapacitor-battery-artificialintelligence.vistec.ac.th) was built as an online tool for any users to classify *CV* or *GCD* based on our model. By simply importing *CV* or *GCD*, the user can determine the confidence of classification as well as the tendency of the material to behave as battery or pseudocapacitor.

8.1 Home page

The Home Page consists of short introduction of the concept of the website as well as the objective of website, graphical demonstration of the ideal of our classification, and navigation bar that provides links to other pages including T.B Robot Page that will provide CV/GCD classifier, About us Page, and Contact us Page as shown in **Supplementary Figure 13**.

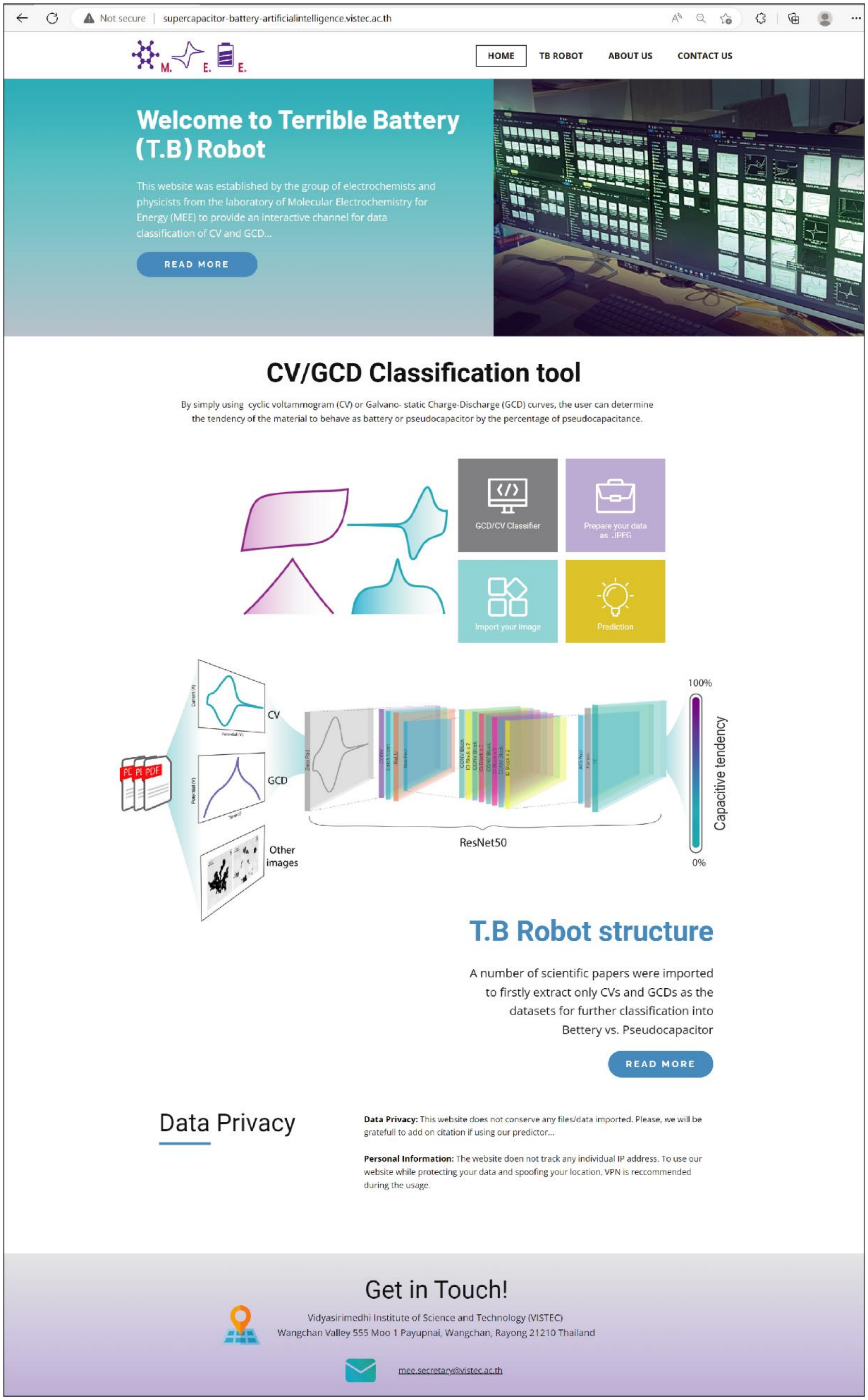
Not secure | supercapacitor-battery-artificialintelligence.vistec.ac.th
M. E. E.
HOME
TB ROBOT
ABOUT US
CONTACT US
Welcome to Terrible Battery (T.B) Robot
This website was established by the group of electrochemists and physicists from the laboratory of Molecular Electrochemistry for Energy (MEE) to provide an interactive channel for data classification of CV and GCD...
READ MORE
CV/GCD Classification tool
By simply using cyclic voltammogram (CV) or Galvano- static Charge-Discharge (GCD) curves, the user can determine the tendency of the material to behave as battery or pseudocapacitor by the percentage of pseudocapacitance.
GCD/CV Classifier
Prepare your data as .JPEG
Import your image
Prediction
CV
GCD
Other images
PDF
ResNet50
100%
0%
Capacitive tendency
T.B Robot structure
A number of scientific papers were imported to firstly extract only CVs and GCDs as the datasets for further classification into Bettery vs. Pseudocapacitor
READ MORE
Data Privacy
Data Privacy: This website does not conserve any files/data imported. Please, we will be gratefull to add on citation if using our predictor...
Personal Information: The website doen not track any individual IP address. To use our website while protecting your data and spoofing your location, VPN is reccommended during the usage.
Get in Touch!
Vidyasirimedhi Institute of Science and Technology (VISTEC)
Wangchan Valley 555 Moo 1 Payupnai, Wangchan, Rayong 21210 Thailand
mee.secretary@vistec.ac.th

Supplementary Figure 13 | The Home page of the website for *CV/GCD* classification.

8.2 T.B Robot page

This page consists of the idea of T.B Robot that is merged from well-established pseudocapacitance concept. Moreover, matching results predicted by our model was demonstrated on this page as well as CNNs concept. From this page, the user can click on ‘GCD’ or ‘CV’ button to start the classifications.

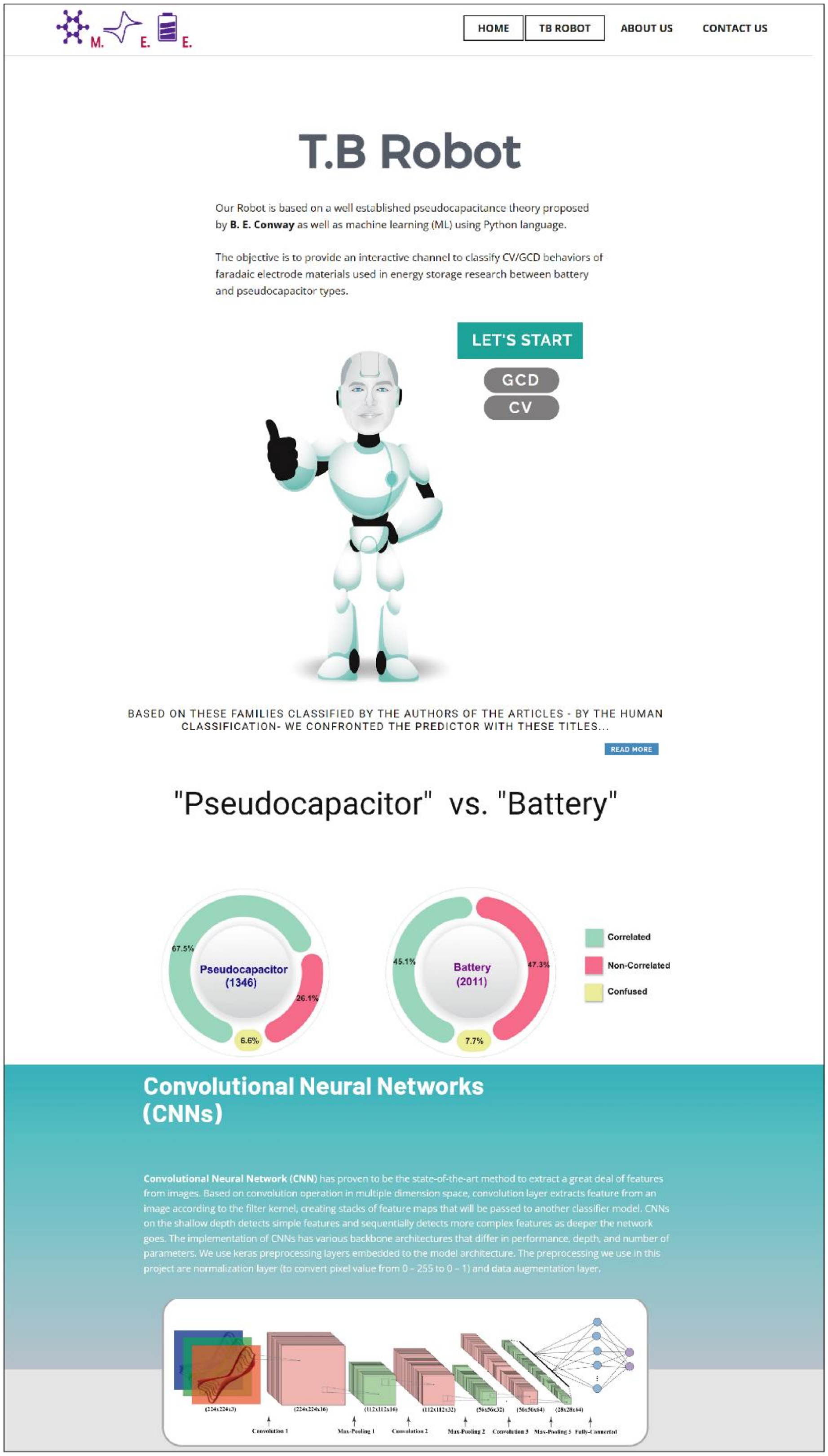

Supplementary Figure 14 | T.B Robot page

8.2.1 *GCD* classification page

This page consists of web application for classifying *GCD* into battery or pseudocapacitor types where the prediction result will appear with the value of % confidence that the picture is battery or pseudocapacitor.

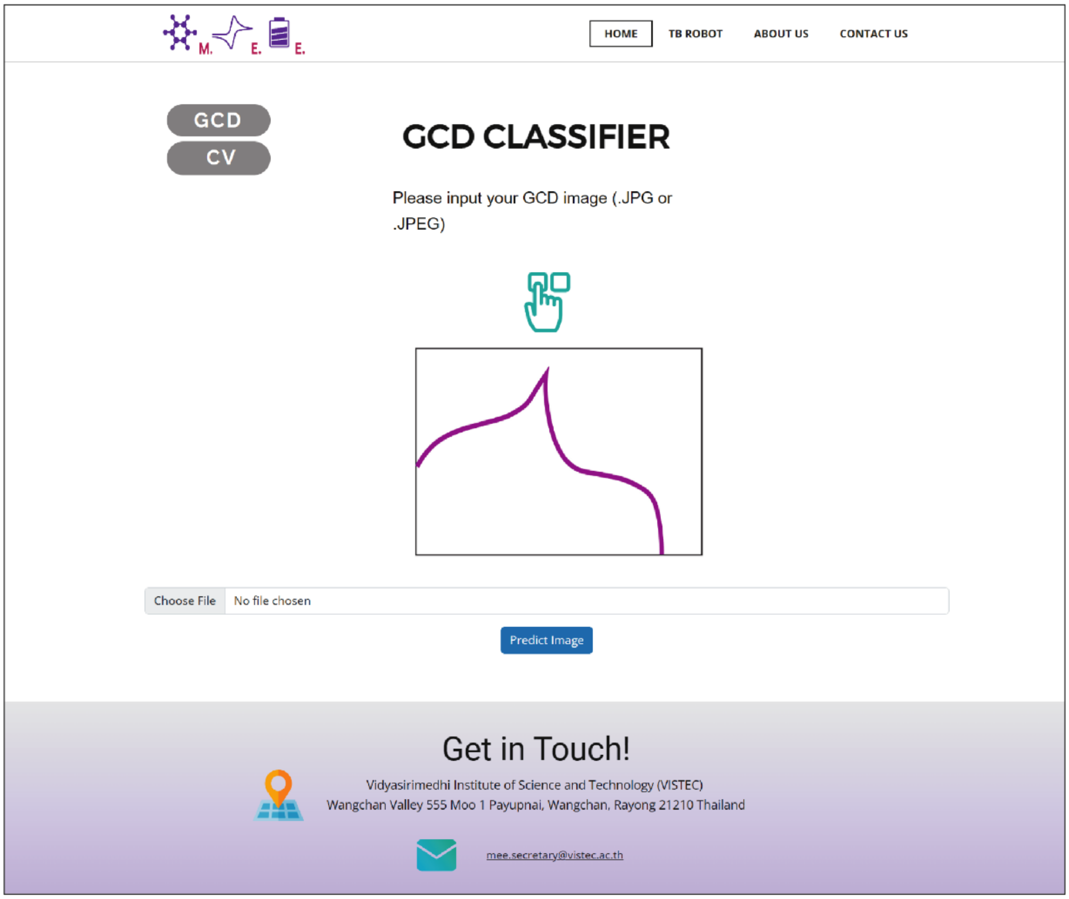

Supplementary Figure 15 | *GCD* classification page

8.2.2 *CV* classification page

This page consists of web application for classifying *CV* into battery or pseudocapacitor types where the prediction result will appear with the value of capacitive tendency of an input *CV*.

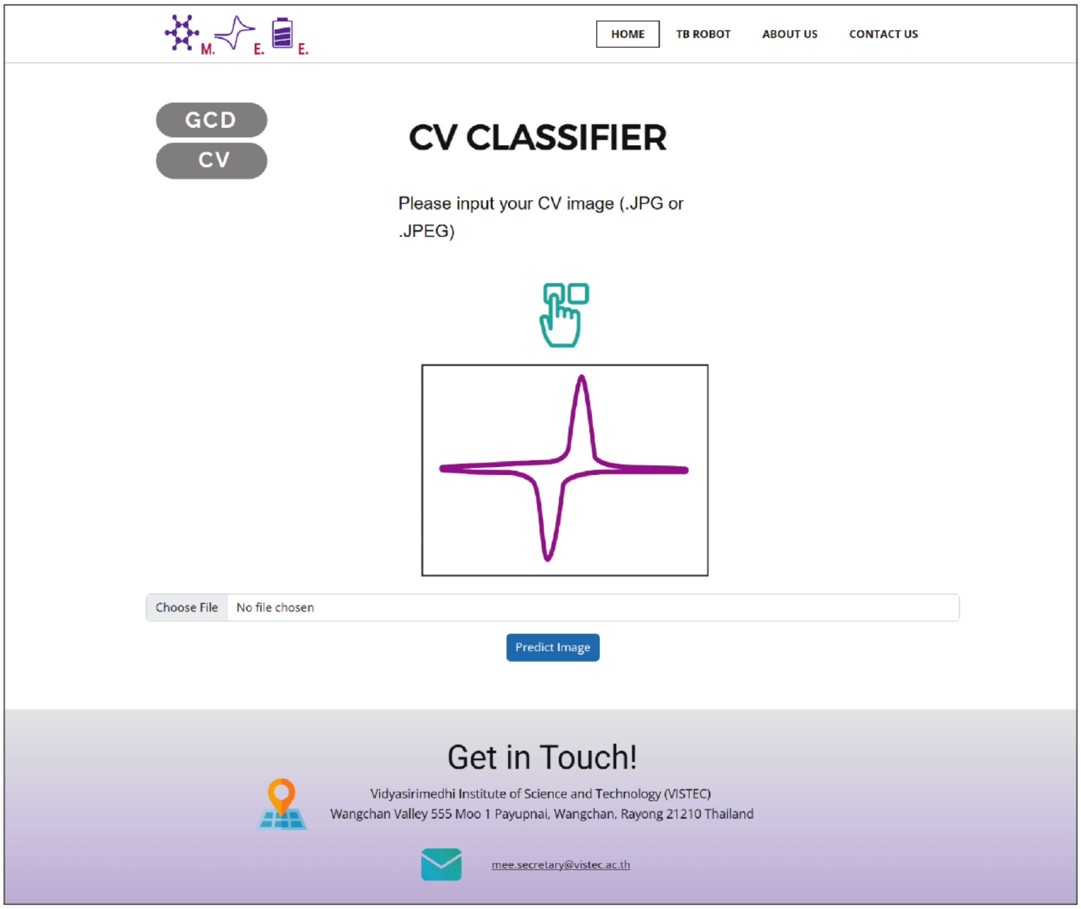

Supplementary Figure 16 | *CV* classification page

8.3 Special issues

The possible issues under the usage of our model are clarified in this section where random pictures besides *GCD/CV* were input in the model. The predicted results with either % confidence or capacitive tendency from those unrelated inputs are demonstrated as shown in **Supplementary Figure 17-20**.

8.3.1 *GCD* classification

Supplementary Figure 17 and 18 show two pictures classified as if it is pseudocapacitor with 84.80% confidence, and battery with 93.93% confidence, respectively. This result could be explained by the reason that this model based on binary classification where any type of input picture can give the prediction as either 1 or 0 (pseudocapacitor or battery), not else. The consequence is that the predictor finds its way to predict with certain value of confidence.

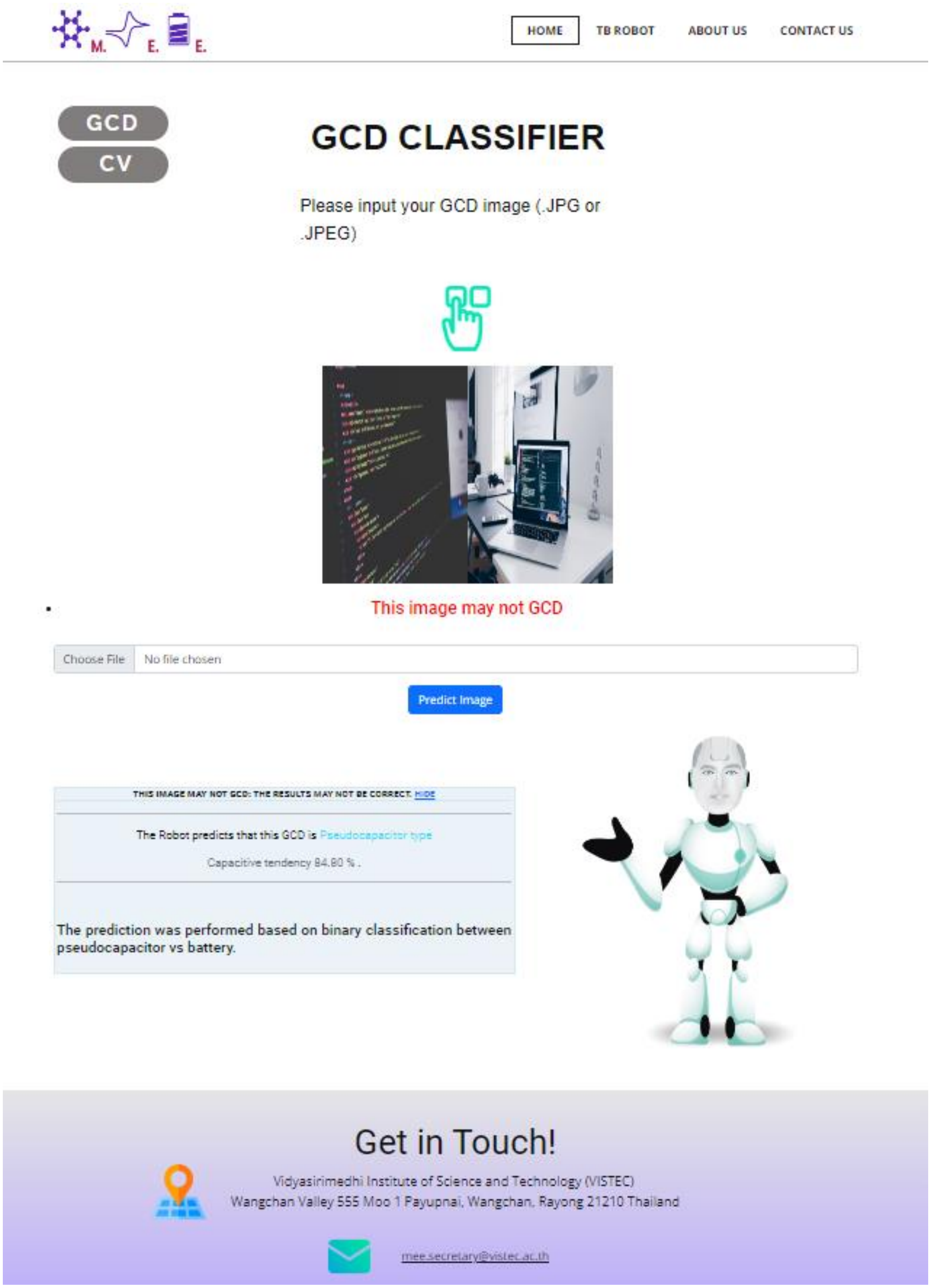

Supplementary Figure 17 | Non-*GCD* input with the prediction as pseudocapacitor

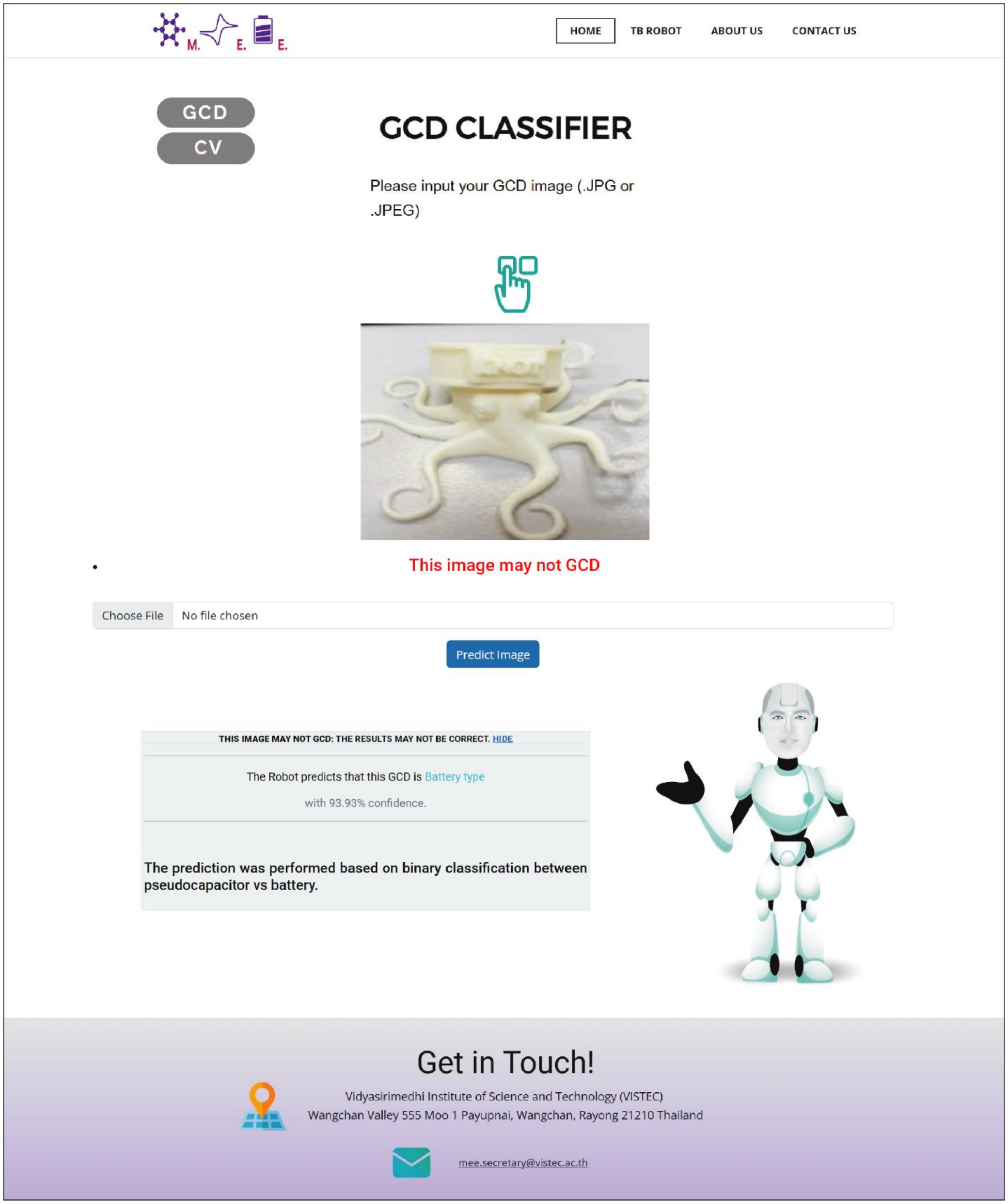

Supplementary Figure 18 | Non-*GCD* input with the prediction as battery.

8.3.2 *CV* classification

Supplementary Figure 19 and 20 show two pictures classified as if it is pseudocapacitor with 96.19 capacitive tendency, and battery with 26.24 capacitive tendency, respectively. This result could be explained by the reason that this model based on binary classification where any type of input picture can give the prediction as either 1 or 0 (pseudocapacitor or battery). The consequence is that the predictor finds its way to predict with certain value of capacitive tendency.

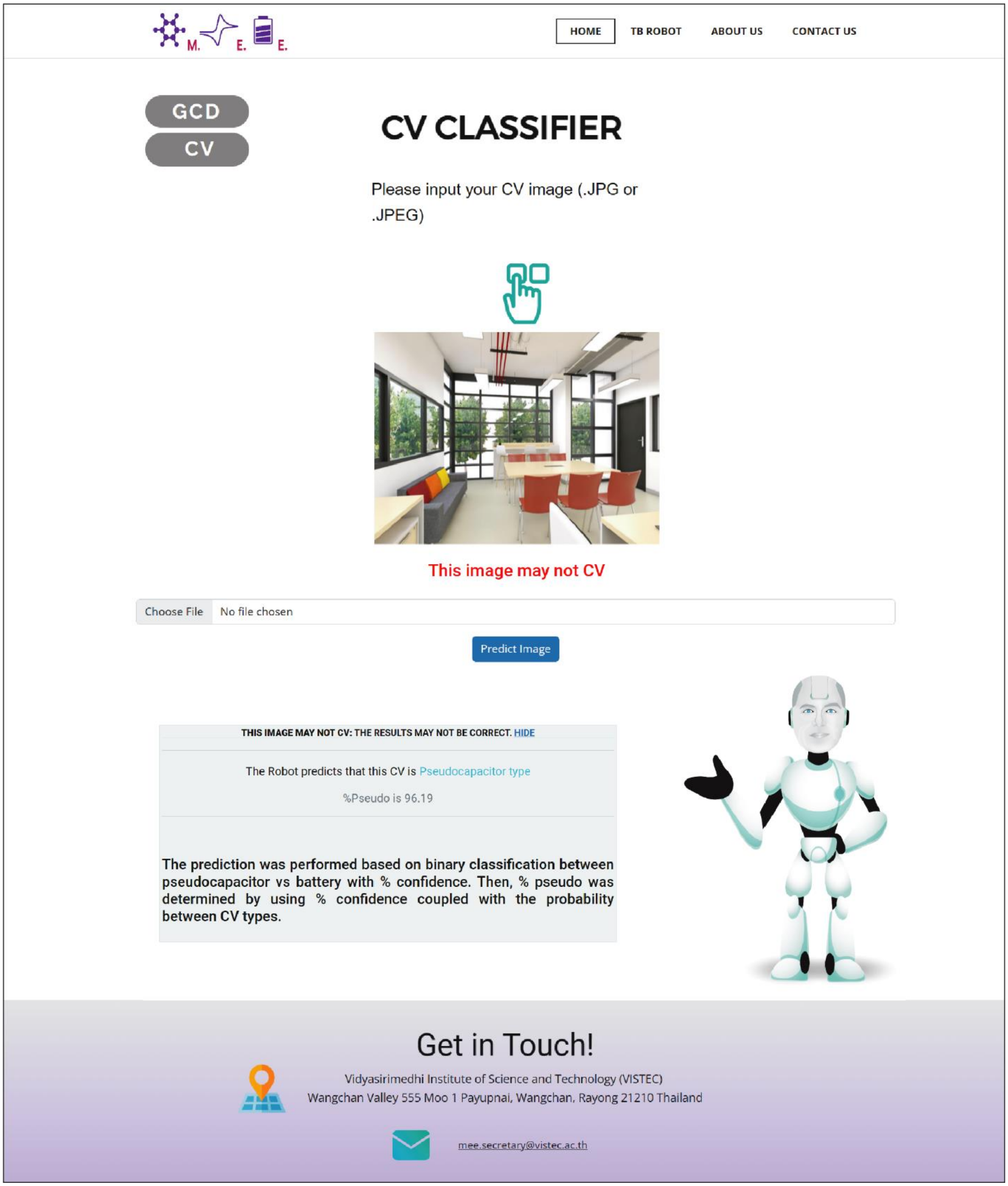

344

345 **Supplementary Figure 19 | Non-*CV* input with the prediction as a pseudocapacitor.**

346

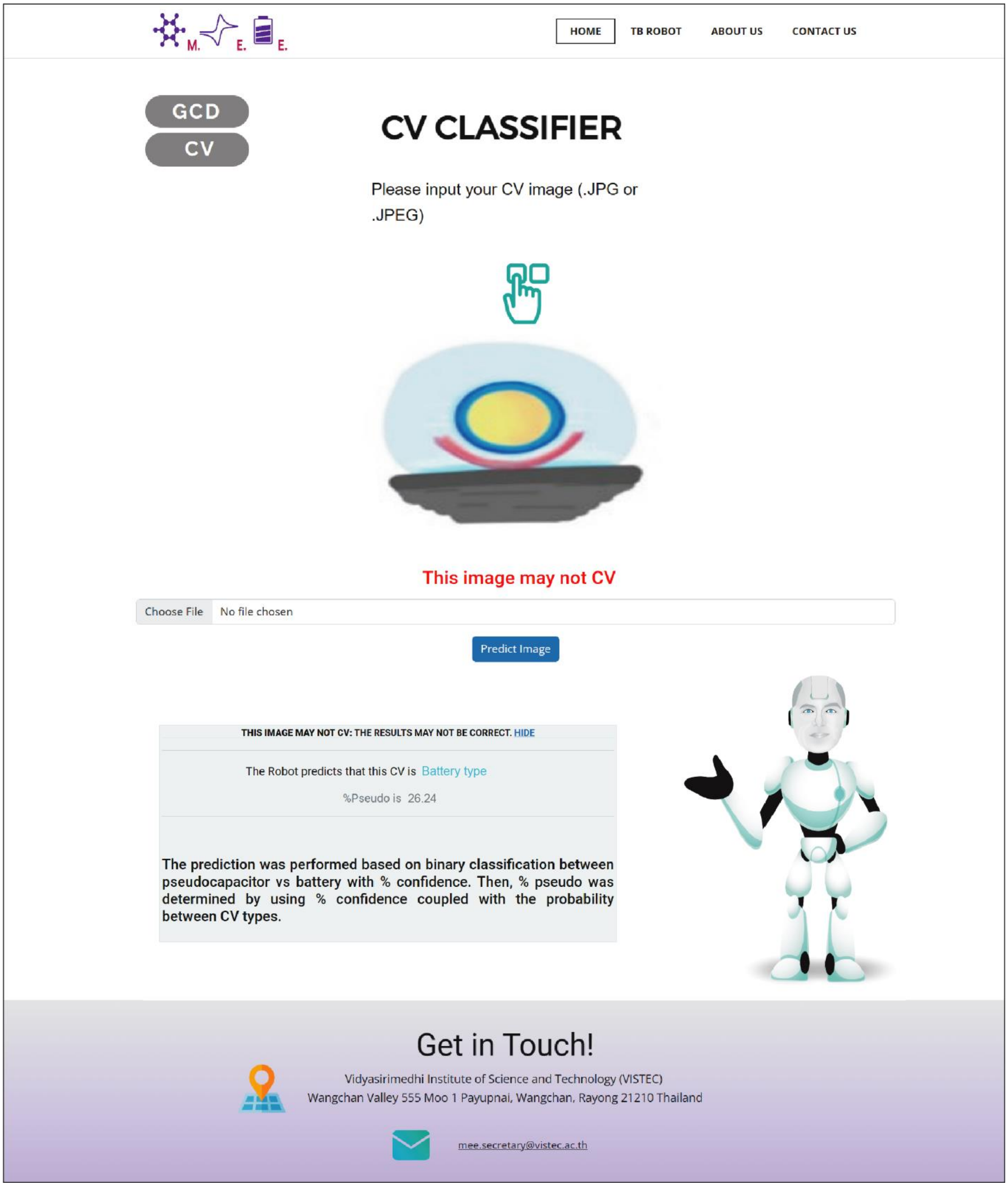

Supplementary Figure 20 | Non-*CV* input with the prediction as a battery.

9. The comparison of literatures of the capacitive prediction

Here, we compared the method from the previous studies using the popular $v/v^{1/2}$ scan rate diagnosis and the predicted result using machine-learning in our study in Supplementary Table 3. The results clearly demonstrate the big difference such in the case of ref [14] and ref [19] that the previous approach from literature ($v/v^{1/2}$ scan rate) gave high percentage capacitance, whereas our study suggested the lower percentage of capacitive tendency. Our method suggests that the capacitive tendency in these cases should be a small number since the peak characteristic of CV is dominant. The limitation of the conventional method that does not cover some situations as pointed in some references [8, 9]. Unlike the conventional model which relies on a proportionality to scan rate, the present capacitive tendency is a geometric variable that does not indicate whether a dynamic is surface-related or diffusion-related. It is based on an analysis of the signal's shape. It should be noted that the historical concept of pseudocapacitance focuses on this geometric shape more than on a surface vs. diffusion dynamic. Through our approach, we propose to researchers a different indicator, one that is more focused on the pure and initial definition.

Reference	Input CV for the classification	Capacitive contribution with scan rate	Capacitive tendency
[10]	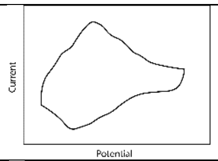	67%	51.90 %
[11]	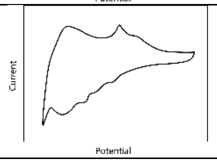	64%	52.04 %

[12]	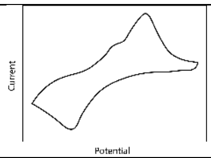	74%	38.27 %
[13]	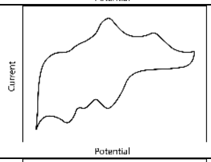	78%	51.94 %
[14]	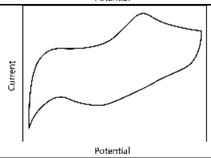	64%	51.94 %
[15]	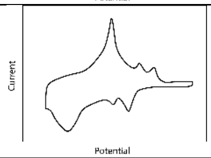	n/a	52.10 %
[16]	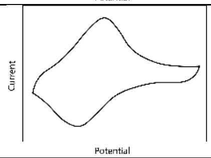	n/a	51.92 %
[17]	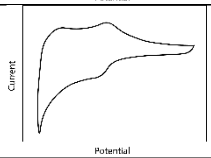	66%	51.90 %
[18]	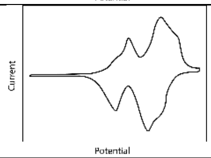	70%	18.26 %
[19]	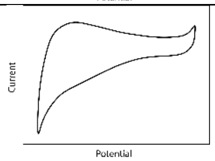	63%	96.03 %
[20]	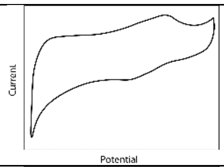	n/a	96.10 %
[21]	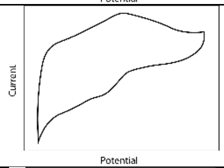	93%	95.80 %
[22]	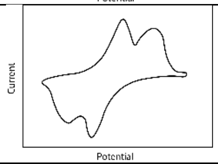	66%	63.45 %

[23]	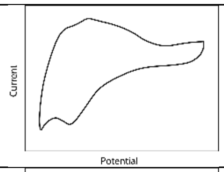	93%	51.93 %
[24]	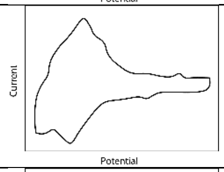	n/a	51.90 %
[25]	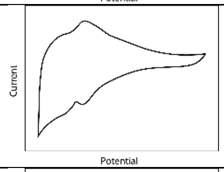	n/a	51.97 %
[26]	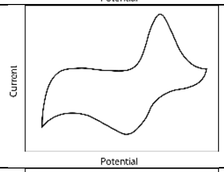	80%	51.98 %
[27]	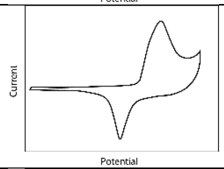	n/a	3.80 %
[28]	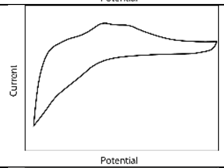	75%	95.47 %
[29]	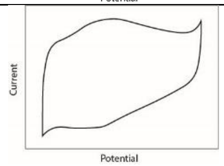	n/a	96.20 %

Supplementary References

1. He, K., et al., *Deep Residual Learning for Image Recognition*. 2016. 770-778.
2. Chollet, F. *Xception: Deep Learning with Depthwise Separable Convolutions*. in *2017 IEEE Conference on Computer Vision and Pattern Recognition (CVPR)*. 2017.
3. Simonyan, K. and A. Zisserman *Very Deep Convolutional Networks for Large-Scale Image Recognition*. 2014. arXiv:1409.1556.
4. Sandler, M., et al., *MobileNetV2: Inverted Residuals and Linear Bottlenecks*. 2018. 4510-4520.
5. Yamashita, R., et al., *Convolutional neural networks: an overview and application in radiology.* Insights into Imaging, 2018. **9**(4): p. 611-629.
6. Lever, J., M. Krzywinski, and N. Altman, *Classification evaluation.* Nature Methods, 2016. **13**(8): p. 603-604.

7. Zhang, Z. and M.R. Sabuncu. *Generalized Cross Entropy Loss for Training Deep Neural Networks with Noisy Labels*. in *NeurIPS*. 2018.
8. Deeba, R., et al., *Controlled Potential Electrolysis: Transition from Fast to Slow Regimes in Homogeneous Molecular Catalysis. Application to the Electroreduction of CO2 Catalyzed by Iron Porphyrin.* ChemElectroChem, 2023. **10**(20): p. e202300350.
9. Guillemin, T., et al., *In-Depth Investigation of Manganese Dioxide as Pseudocapacitive Electrode in Lithium- and Sodium-Doped Ionic Liquids.* Journal of The Electrochemical Society, 2023. **170**(10): p. 100531.
10. Shao, H., et al., *Electrochemical study of pseudocapacitive behavior of Ti3C2Tx MXene material in aqueous electrolytes.* Energy Storage Materials, 2019. **18**: p. 456-461.
11. Hu, L., et al. *Cu2Se Nanoparticles Encapsulated by Nitrogen-Doped Carbon Nanofibers for Efficient Sodium Storage*. Nanomaterials, 2020. **10**, DOI: 10.3390/nano10020302.
12. Jiang, Y. and J. Liu, *Definitions of Pseudocapacitive Materials: A Brief Review.* Energy & Environmental Materials, 2019. **2**(1): p. 30-37.
13. Hu, L. and C. Shang *Co3V2O8 Nanoparticles Supported on Reduced Graphene Oxide for Efficient Lithium Storage*. Nanomaterials, 2020. **10**, DOI: 10.3390/nano10040740.
14. Costentin, C., *Electrochemical Energy Storage: Questioning the Popular v/v1/2 Scan Rate Diagnosis in Cyclic Voltammetry.* The Journal of Physical Chemistry Letters, 2020. **11**(22): p. 9846-9849.
15. Zhang, J., et al., *Urchin-Like Fe3Se4 Hierarchitectures: A Novel Pseudocapacitive Sodium-Ion Storage Anode with Prominent Rate and Cycling Properties.* Small, 2020. **16**(26): p. 2000504.
16. Mishra, N.K., R. Mondal, and P. Singh, *Synthesis, characterizations and electrochemical performances of anhydrous CoC2O4 nanorods for pseudocapacitive energy storage applications.* RSC Advances, 2021. **11**(54): p. 33926-33937.
17. Liu, X., et al., *Ultrafine MoO3 nanoparticles embedded in porous carbon nanofibers as anodes for high-performance lithium-ion batteries.* Materials Chemistry Frontiers, 2019. **3**(1): p. 120-126.
18. Chong, S., et al., *Potassium Nickel Iron Hexacyanoferrate as Ultra-Long-Life Cathode Material for Potassium-Ion Batteries with High Energy Density.* ACS Nano, 2020. **14**(8): p. 9807-9818.
19. Wang, G., et al., *Hierarchical Carbon Nanosheet Assembly with SiOx Incorporation and Nitrogen Doping Achieves Enhanced Lithium Ion Storage Performance.* Advanced Energy and Sustainability Research, 2021. **2**(7): p. 2100026.
20. Zhang, W., et al., *Mesoporous TiO2/TiC@C Composite Membranes with Stable TiO2-C Interface for Robust Lithium Storage.* iScience, 2018. **3**: p. 149-160.
21. Chen, H., et al., *A new spinel high-entropy oxide (Mg0.2Ti0.2Zn0.2Cu0.2Fe0.2)3O4 with fast reaction kinetics and excellent stability as an anode material for lithium ion batteries.* RSC Advances, 2020. **10**(16): p. 9736-9744.
22. Zhang, C., et al., *Polyimide@Ketjenblack Composite: A Porous Organic Cathode for Fast Rechargeable Potassium-Ion Batteries.* Small, 2020. **16**(38): p. 2002953.
23. Xu, W., et al., *Sn nanocrystals embedded in porous TiO2/C with improved capacity for sodium-ion batteries.* Inorganic Chemistry Frontiers, 2019. **6**(10): p. 2675-2681.
24. Wei, T., et al., *An electrochemically induced bilayered structure facilitates long-life zinc storage of vanadium dioxide.* Journal of Materials Chemistry A, 2018. **6**(17): p. 8006-8012.
25. Li, H., et al., *A High-Performance Sodium-Ion Hybrid Capacitor Constructed by Metal–Organic Framework–Derived Anode and Cathode Materials.* Advanced Functional Materials, 2018. **28**(30): p. 1800757.
26. Li, S., et al., *Encapsulation of MnS Nanocrystals into N, S-Co-doped Carbon as Anode Material for Full Cell Sodium-Ion Capacitors.* Nano-Micro Letters, 2020. **12**(1): p. 34.

27. Ren, C., et al., *Hierarchical Porous Integrated Co1−xS/CoFe2O4@rGO Nanoflowers Fabricated via Temperature-Controlled In Situ Calcining Sulfurization of Multivariate CoFe-MOF-74@rGO for High-Performance Supercapacitor.* Advanced Functional Materials, 2020. **30**(45): p. 2004519.
28. Jia, H., et al., *Advanced ZnSnS3@rGO Anode Material for Superior Sodium-Ion and Lithium-Ion Storage with Ultralong Cycle Life.* ChemElectroChem, 2019. **6**(4): p. 1183-1191.
29. Gong, Y., et al., *Electric Double-layer Capacitance and Pseudocapacitance Contributions to the Oxidative Modification of Helical Carbon Nanofibers.* International Journal of Electrochemical Science, 2020. **15**(8): p. 7508-7519.
